# Reflections on end-of-life dialysis

**DOI:** 10.1590/2175-8239-JBN-3833

**Published:** 2018-05-17

**Authors:** Manuel Carlos Martins Castro

**Affiliations:** 1 Universidade de São Paulo, Faculdade de Medicina, Hospital das Clínicas, São Paulo, SP, Brasil.

**Keywords:** dialysis, quality of life, palliative care, kidney failure, chronic, advanced care planning, diálise renal, qualidade de vida, cuidados paliativos, insuficiência renal crônica, planejamento antecipado de cuidados

## Abstract

The world population is aging and diseases such as diabetes mellitus and systemic arterial hypertension are increasing the risk of patients developing chronic kidney disease, leading to an increase in the prevalence of patients on dialysis. The expansion of health services has made it possible to offer dialysis treatment to an increasing number of patients. At the same time, dialysis survival has increased considerably in the last two decades. Thus, patients on dialysis are becoming more numerous, older and with greater number of comorbidities. Although dialysis maintains hydroelectrolytic and metabolic balance, in several patients this is not associated with an improvement in quality of life. Therefore, despite the high social and financial cost of dialysis, patient recovery may be only partial. In these conditions, it is necessary to evaluate the patient individually in relation to the dialysis treatment. This implies reflections on initiating, maintaining or discontinuing treatment. The multidisciplinary team involved in the care of these patients should be familiar with these aspects in order to approach the patient and his/her relatives in an ethical and humanitarian way. In this study, we discuss dialysis in the final phase of life and present a systematic way to address this dilemma.

## RESUMO

A população mundial está envelhecendo, e doenças como diabetes mellitus e hipertensão arterial sistêmica estão aumentando o risco de doença renal crônica, com consequente elevação na prevalência de pacientes em diálise. A expansão dos serviços de saúde permitiu oferecer tratamento dialítico para um número cada vez maior de pacientes. Paralelamente, a sobrevida em diálise aumentou consideravelmente nas últimas duas décadas. Dessa maneira, os pacientes em diálise são cada vez mais numerosos, mais idosos e com maior número de comorbidades. Embora a diálise mantenha o equilíbrio hidroeletrolítico e metabólico, em diversos pacientes isso não está associado à melhora da qualidade de vida. Então, apesar do elevado custo social e financeiro da diálise, a recuperação do paciente pode ser apenas parcial. Nessas condições, é necessário avaliar individualmente o paciente em relação ao tratamento dialítico, o que implica reflexões sobre iniciar, manter ou suspender o tratamento. A equipe multidisciplinar envolvida no cuidado desses pacientes deve estar familiarizada com esses aspectos para abordar o paciente e seus familiares de forma ética e humanitária. Neste estudo, foi discutido o tratamento dialítico na fase final da vida e apresentada uma maneira sistemática para enfrentar esse dilema.

## INTRODUCTION

Following the diagnosis of chronic renal failure, there is a phase of intense treatment, in an attempt to block or reduce its progression to dialytic renal disease. The attempts will be of greater or lesser intensity, according to the disease stage upon presentation. Over time, there is a defervescence in the measures for primary control of the disease and there is an increase in supportive care or palliative treatment.^1^

In more advanced stages, usually when the prognosis is of less than six months, the patient may enter a phase in which there is a need for care in rear units or intensive home care, often followed by death and mourning.

**Figure 1:**
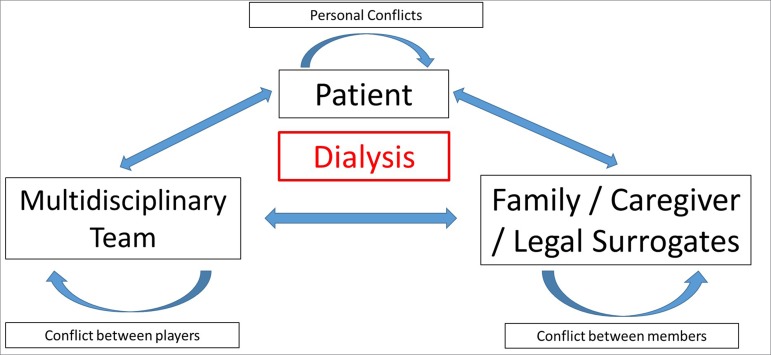
Associations and interdependence among the different segments involved in dialysis.

In the dialysis treatment onset phase, at least three variables must be considered: the patient; the family, the caregiver or the legal guardian; and the multiprofessional team. Each of these variables can present conflicts, whether personal, between family members, between caregivers and the legal guardian, or even among members of the multiprofessional team. These three vertices of the problem can exert influences on each other, turning decision making even more complex and difficult (Figure 1).

### WHAT TO CONSIDER WHEN DIALYSIS TREATMENT IS PRESCRIBED

When a patient goes to start chronic dialytic treatment, some considerations must be made. Perhaps the main and most important is the answer to a question that involves medical and ethical aspects: will dialysis increase the patient's time and quality of life or simply prolong the death process?

The problem is not only limited to the beginning of dialysis. Often, discontinuing dialysis can be as difficult a decision as initiating treatment. So, a similar reasoning can be applied to the patient who is already undergoing dialysis, whose clinical evolution is not being adequate: is dialysis increasing the patient's time and quality of life or simply prolonging the death process?

Therefore, it is necessary to analyze and balance the concepts of quality and quantity of life - which are subjective and can vary over time, especially when the degree of recovery provided by the treatment instituted is inadequate. Often, an acute event or other serious illness is necessary to not indicate or stop dialysis.

### ASSESSMENT OF THE RISK OF DEATH ON DIALYSIS

Several instruments have been proposed and used to evaluate the risk of death in patients undergoing dialysis. One of the simplest, most effective and most used is based on the answer to the question: "Would you be surprised if that patient died in the next twelve months?" Two answers fit the question: 1) yes, I would; and 2) no, I would not be surprised.^2^

The study by Moss *et al*., published in 2008, involved 147 patients on hemodialysis in three different units, showed that the risk of death was 3.5 times higher when the answer to the question was "no, I would not be surprised" (p = 0.01).^2^ Cohen *et al*, in a study published in 2010, encompassing 512 patients undergoing hemodialysis at five clinics, also using the negative response to the question: "Would you be surprised if this patient died in the next six months? ", they showed that the answer" no, I would not be surprised "increased the risk of death by 2.7 times.^3^

Therefore, the simple answer to this question can be a powerful tool to evaluate the risk of mortality in hemodialysis. However, the question poses a high degree of subjectivity, since the answer will depend on the observer's own experience.

Other variables such as age^4^, serum albumin,^5^ Charlson's comorbidity index,^6^ and Karnofsky's performance scale^7^ have also been used to assess mortality risk in dialysis patients. In the study by Moss *et al*., patients, for whom the answer to the question was "no, I would not be surprised", had significantly higher age and lower Charlson comorbidity index and serum albumin and Karnofsky's performance scale than the patients on whom the answer was: "yes, I would be surprised."^2^

Cohen *et al*. demonstrated in their study that not only the surprise question, but also the reduction of serum albumin and increased age, in addition to the diagnoses of peripheral vascular disease and dementia, were associated with an increased risk of mortality.^3^

These observations confirm the usefulness of the surprise question in assessing the mortality risk. However, depending on the observer's experience, false-positive and false-negative results will be common, causing variations in the sensitivity and specificity of this instrument to assess the risk of death on dialysis.

In 2009, Couchoud *et al*. published a clinical score to predict the six-month prognosis in elderly patients over 75 years of age and chronic kidney disease, initiating dialysis.^8^ The index was composed of nine risk factors: body mass index (≥ 18.5 or < 18.5 kg/m^2^), presence or absence of diabetes mellitus, congestive heart failure, peripheral vascular disease, arrhythmias, active neoplasia, severe behavioral disorder, total dependence for locomotion and the context of dialysis onset (planned or unplanned). The authors reported that, in the index validation population, patients with scores 0, 1, 2, 3 to 4, 5 to 6, 7 to 8 and ≥ 9 had mortality rates of 8, 10, 17, 21, 33, 50 and 70%, respectively. In patients with index ≥ 7, the dialysis suspension was the cause of death in 15% of the cases.^8^

Nowadays, calculator websites are available to predict the mortality risk of hemodialysis patients, providing information such as age, serum albumin, with or without dementia or peripheral vascular disease, and the answer to the surprise question. The program estimates the expected survival for 6, 12 and 18 months (touchcalc.com/calculation/sq).

In this review, more important than an index that effectively evaluates the risk of death, is an index that enables establishing a strategy regarding the preparation of the patient, family, caregiver, legal guardian and multiprofessional team in relation to future events, and indicate how to conduct each case within the established treatment planning.

## TREATMENT OPTIONS FOR ADVANCED CHRONIC KIDNEY DISEASE

From stage 4 chronic kidney disease, glomerular filtration rate less than 30ml/min, the clinician or family physician in association with a nephrologist physician are authorized to begin planning for future renal replacement therapy. Two conditions are possible: 1) maintenance of the conservative treatment to the end of life, and 2) renal function replacement therapy through dialysis or renal transplantation.

In any option, for patients at higher risk of death, it is necessary to begin the preparation of an advanced care plan, a palliative care plan and an end-of-life care plan that includes planning in the terminal care phase.

### THE "GOOD DEATH" CONCEPT

The concept of good death is broad. Generally, death is considered a good death when it happens without pain, brief, in peace, without avoidable suffering for the patient, the family and the caregiver, in the company of the loved ones and in the place that the patient chose to die. Within this process, the local medical, cultural and ethical standards must be respected.^9^^-^ 13

The main barriers to a good death are: inadequate control of pain and other symptoms; emotional stress for the patient and the family; lack of attention to family dynamics; lack of knowledge of the patient and the family regarding end-of-life care and lack of an advanced care plan.^14^^,^ 15

It is therefore possible to conclude that a bad death is accompanied by unnecessary suffering, at odds with the wishes of the patient and the family, having a feeling that the norms of decency have been faced. In that sense, an advanced care plan helps reduce possibilities for the patient to have a bad death.

### ADVANCED CARE PLAN

The advanced care plan aims to establish a process of communication between the patient, the family, the health care team, and other important people regarding the patient's wishes for end-of-life care. The main goal is to enable patients to have control over their health care, preparing both, the patient and the family, for a good death.^15^^,^ 16

The advanced care plan implementation should be initiated when the health care team answers that they would not be surprised if the patient died in the following 12 months.

Before the advanced care plan is implemented, it is critical to evaluate the patient in relation to eventual cognitive impairment. In addition, conditions such as anxiety, depression and fear tend to lower the pain threshold, and cause some confusion as to when to start the advanced care plan.^15^

The main care plan has as main attributes:

1. Expand the patient's and family's knowledge about terminal chronic kidney disease in relation to end-of-life aspects and care options;
2. Identify the patient's priorities for end-of-life care and develop a plan of action;
3. Identify the person who will take over and participate in medical decisions in case of patient incapacity;
4. Help the person in charge to understand his/her importance;
5. Prepare the patient and family for death;
6. Enable the patient to have control over his/her health care; and
7. Relieve the burden on loved ones by strengthening interpersonal relationships.

### PALLIATIVE CARE PLAN

The palliative care plan aims to improve the quality of life of the patient and family in the face of a fatal illness.^17^^,^ 18 This is done by preventing and alleviating suffering resulting from early identification, evaluation and treatment of pain and as well as physical, psychological and spiritual problems. It is highly recommended that palliative care be extended to caregivers and remains active during mourning.^19^

The palliative care plan does not exclude the presence of an active treatment. In the specific case of chronic end-stage renal disease, it should be made available for patients who have chosen conservative treatment, those who have decided to stop dialysis, and those who have decided to maintain dialysis treatment.^15^ Palliative care can be offered in the hospital, clinics or backup hospitals, or at the patient's home.

The main objectives of a well-structured palliative care plan are:

1. Relieve pain and other distressing symptoms;
2. To regard life and death as a normal and natural process;
3. Do not hasten or delay death;
4. Integrate psychological and spiritual aspects in patient care;
5. Guarantee support in the family process of coping with the illness and the period of mourning;
6. Provide a multiprofessional team to meet the needs of the patient and the family, including the mourning period;
7. To improve the quality of life, seeking to positively influence the course of the disease; and
8. Understand and better manage distressing clinical complications, either alone or in combination with other conventional or non-conventional treatments.

The palliative care plan should be developed by a palliative care physician using trained providers to specifically assist patients who need this type of care. In the absence of these professionals, caregivers who are not specialized in the field may offer the service after receiving adequate training and instruction.

### END-OF-LIFE CARE AND TERMINAL CARE PLAN

The end-of-life care plan aims to provide patients with progressive, incurable diseases, such as terminal chronic kidney disease, care that will enable them to live as well as possible until death.^15^ This action should be complemented with a plan for end-of-life care that aims to offer the patient, in the last few days or weeks of his life, comfort and symptom relief, enabling the family and patient to bid farewell.^15^ The primary objective of the end-of-life care plan and the terminal care plan is to prepare and offer a good death.^18^

### SUPPORTIVE AND SPIRITUAL CARE

Supportive care is non-medical care aimed at helping patients cope with the diagnosis of chronic end-stage renal disease, so that they can express and understand their emotions. This measure enables the patient to be strengthened through the power of control and choice.^15^^,^ 18 Often, especially in developing countries, it is necessary to include financial support for the patient and the family. Finally, spiritual care should be offered to meet the needs of the patient, helping them deepen their faith regardless of religious belief.^15^^,^ 18

### REFLECTIONS FOR THE PHYSICIAN ON THE DEATH PROCESS

It is critical for the multiprofessional team, and particularly for the attending physician, to understand that caring for those who are dying is an integral and important part of health care,^9^ which should involve and respect the patient and all who are close to him.

Undoubtedly, for the physician to offer the patient who is dying a good end-of-life care, it is necessary to have interpersonal skills, clinical knowledge, technical support, information based on scientific evidence, personal and professional values and experience. In this sense, changing the vision and culture of an organization is a great challenge, but often a condition for individual change. Therefore, healthcare professionals have a special responsibility to educate themselves in the processes of identification, management and discussion about the final phase of a fatal illness.^9^

More comprehensive studies in the future will be needed to expand clinical, cultural and organizational knowledge, as well as to develop learning that will incorporate different practices that can minimize the suffering of those who are dying.

The burnout syndrome refers to a condition of physical and mental exhaustion, with a depressive aspect to it, closely related to professional life, which mainly affects healthcare professionals, such as doctors, nurses, physiotherapists, social workers and nutritionists.^20^ Daily coexistence with the suffering of others generates a kind of defense mechanism, and the professional tends to become less sensitive to physical and spiritual pain. However, there cannot be absolute insensibility, since it is not in accord with the primary function of medicine.

### CONSERVATIVE TREATMENT IN CHRONIC KIDNEY DISEASE

In stage 5 chronic kidney disease, a glomerular filtration rate of less than 15 ml/min, is indicative of the need to initiate renal replacement therapy, conservative treatment is defined as the set of actions and care offered to the patient that do not include dialysis or renal transplantation.

The decision not to initiate replacement renal therapy may be made by the patient himself, when cognitive conditions enables such a decision, or by a family member or legal guardian previously vested with that authority.

Conservative treatment is a holistic planning centered on the patient with stage 5 chronic kidney disease, which actions aim at:

1. Delay progression and minimize complications and adverse events;
2. Share decisions;
3. Manage symptoms;
4. Detailed communication, including advanced care plan;
5. Psychological support;
6. Social and family support; and
7. Attention to cultural and spiritual aspects.

Conservative treatment of stage 5 chronic kidney disease should begin early, when it is intended to provide quality treatment to patients who have not benefited from dialysis or have not opted for it.^21^ The team involved in providing these services should be multiprofessional, composed of: physician nephrologist, family doctor, nurse, social worker, psychologist, nutritionist and a religious and spiritual support service. All staff members must have training, expertise, and availability for care in the hospital, the back-up hospital, nursing homes, or the patient's home.

Experience has shown that after the introduction of a structured plan for conservative treatment of chronic kidney disease, the number of hospital admissions, visits to emergency units and ICU admissions decreases; hospitalizations are being carried out more in back-up hospitals; the 30-day rehospitalization rate is lower; the number of deaths in intensive care units is lower; and consequently reduces treatment cost.^1^^,^ 22

Finally, it should be noted that, in the case of patients with absolute indication for initiating dialysis, the median survival time in conservative treatment is approximately 6 to 7 months.^23^ In this period, renal treatment should be continuously adjusted according to patient evolution, and the multiprofessional team should initiate the advanced care plan followed by the palliative care plan, as previously established.

### DIALYTIC TREATMENT OF CHRONIC KIDNEY DISEASE

Initiating or discontinuing dialysis treatment should be a shared decision involving the medical staff, the multiprofessional team, the patient, the family, the caregiver and, if appropriate, the legal guardian. Therefore, over time, it is necessary to establish a physician-patient relationship that enables shared decision-making.^24^^,^ 25

For the patient and others involved, being adequately informed is fundamental. In this sense, every patient with stages 4, 5 and 5D chronic kidney disease should be informed about the diagnosis and treatment options and, particularly, for patients in stages 5 and 5D, a prognostic estimate should be offered according to the current clinical condition.^16^^,^ 24 ^,^ 25 Creating an environment conducive to shared decision-making in association with a fully informed patient will enable an advanced care plan.

It will always be possible to consider not initiating or discontinuing dialysis in the treatment of chronic kidney disease when:

1. The decision-making patient voluntarily refuses dialysis or requests that it be discontinued;
2. A patient who, although at a certain moment of evolution does not have full capacity to make decisions, has previously, orally, preferably written, refused to start dialysis or asked to discontinue it;
3. A patient who, without decision-making ability, has adequately indicated a legal guardian who refuses or requests that the dialysis be discontinued; and finally,
4. The patient with irreversible and profound neurological damage that is unconscious or does not show signs of sensitivity, intentional behavior and self-awareness and that of the environment.

The decision not to initiate or discontinue dialysis can be made easier for patients who have a very poor prognosis or for whom dialysis cannot be offered safely.^24^^-^ 26 This is the case for patients with inability to understand (advanced dementia, those pull the needles or the dialysis catheter); those with very unstable hemodynamic condition (severe hypotension); those in need for sedation to perform the dialysis procedure; with non-renal terminal disease (consider that some patients in this condition may benefit from choosing to undergo dialysis); and, finally, patients over 75 years of age with chronic kidney disease who have two or more of the following criteria:

1. Negative answer to the surprise question ("no, I would not be surprised if the patient died");
2. Charlson comorbidity index ≥ 8;
3. Acute functional disability with Karnofsky index ≤ 40; and
4. Severe malnutrition with serum albumin < 2.5 g/dl.

Therefore, the use of some additional instruments may help in deciding whether to offer dialysis to a particular patient. An assessment o estimate the presence and degree of depression, the degree of cognitive impairment, the degree of comorbidities (Charlson index), the degree of functional disability (Karnofsky's index), the frequency and severity of symptoms during the dialysis sessions, and a mortality predictor in the next six months, can and should support this decision.

Despite the use of these instruments, there will always be cases where there will be no consensus on what should be done. When this occurs, consideration should be given to providing a limited dialysis time for the patient who presents an uncertain prognosis or for whom a consensus decision has not been made.^24^^,^ 25 This means that it is necessary to establish an action plan for conflict resolution when there is no agreement as to what decision should be made in connection with the dialysis. This plan should include the use of a uniform approach among those involved in the communication about the diagnosis, prognosis, treatment options and objectives.

### RECOMMENDATIONS FOR CLINICAL PRACTICE

#### PLANNING, INITIATING, AND DISCONTINUING DIALYSIS

Every patient with stages 4 and 5 chronic kidney disease should have a prognostic evaluation and an estimate of quality of life with and without dialysis. Whatever the outcome of this evaluation, conservative treatment should be offered to the patient and family, regardless of whether they chose not to initiate, or discontinue dialysis.

In patients with evident clinical worsening, despite dialysis, clinical follow-up will allow to recognize the imminent or immediate need for end-of-life care, regardless of whether or not clinical worsening occurs in the presence of a catastrophic acute event. Therefore, it is imperative to maintain a frequently updated record about supportive care, especially for patients with a life expectancy of less than one year, with the register of comorbidities, functional condition, evidence of malnutrition, cognitive status in cases of advanced age and answer the surprise question.

Outlining the advanced care plan, especially for patients who have chosen conservative treatment or those who are worsening despite dialysis, is essential in order to standardize posture and conduct among multiprofessional team members, the patient and the family, the caregiver, and the legal guardian. Discontinuing dialysis treatment is one aspect to be included in the advanced care plan and a decision to be made within a life-long care plan. This decision should always be implemented in a multidisciplinary environment, involving the patient, the family, the caregiver, the legal guardian, the nephrologist and the family doctor.

Deciding not to start or discontinue dialysis is ethical and clinically acceptable, as long as the process is supported by a shared decision. Conditions that may influence this decision, such as depression, physical pain, and potentially reversible social factors, should be evaluated and controlled. It is prudent and fundamental to emphasize that the decision not to initiate or discontinue dialysis can only be implemented after careful evaluation to exclude diagnoses of depression or burnout syndrome in any of those involved.

After dialysis discontinuation, the patient, the family, the caregiver and the legal guardian must be guaranteed continuation of supportive care and/or palliative care. In end-of-life care, good communication, symptom relief, psychological and spiritual support, tailored to the needs of the patient and family, and, where possible, patient and family care at the place of their choice are actions to address the issue. In addition, it is important to offer a culturally appropriate grief service to the family, caregiver, and legal guardian after the denouement.

Finally, shared decision, advanced care, palliative care, end-of-care and terminal care plans must be updated at least annually or more frequently if necessary. In these plans, the patient should always be properly informed that he has the right to refuse dialysis, even if the medical staff is not in agreement with the decision, while the medical staff must be aware that they also have the right to refuse dialysis when the benefits do not justify the risks, even when the patient or family requests treatment.

### AUDIT TOOLS

Several particularities discussed in this review involve ethical and legal aspects,^27^ therefore, it is appropriate to establish audit parameters that properly evaluate the results and protect the multiprofessional team.^16^ Different indexes may be used to monitor the program's performance, such as:

1. Registry of patients in end-of-life care, including those on conservative treatment, those on dialysis with a worsening clinical condition and those withdrawn from dialysis;
2. Proportion of patients in end-of-life care who died;
3. Proportion of patients with stage 5 chronic kidney disease in supportive treatment compared to the total number of patients on conservative treatment;
4. Proportion of deaths due to suspension of dialysis in relation to total deaths;
5. Proportion of patients in end-of-life care who have an advanced care plan; and
6. Proportion of patients who received end-of-life care at their preferred location.

## FINAL CONSIDERATIONS

Many patients with chronic kidney disease may be kept on conservative treatment, without initiating dialysis, for their best interest. On the other hand, dialysis patients may also benefit from access to supportive care at the outpatient ward, home, back-up hospitals or respite care. However, in any case, for the patient approaching the end of life, offering palliative care becomes essential.

The layman will always have the idea that end-of-life dialysis refers to situations involving, especially, elderly patients. This is not true. Regardless of age, in any individual with end-stage kidney disease, who is progressively getting worse and/or in life-threatening clinical worsening, the aspect of end-of-life dialysis can and should be addressed.

When dialysis is not initiated or stopped, conservative and palliative care programs emerge as strategies for managing chronic dialysis dependence. When such care is not offered to patients with end-stage kidney disease, there is significant suffering, which generates psychosocial burnout to caregivers, the family, and the community. Nephrologists should be familiar with supportive and palliative treatment options, understanding them as part of their professional responsibility.

Physicians have a duty to provide the patient and all decision-makers with sufficient information about treatment options. This means explaining all the treatment modalities available, with their benefits and harms, and the types and consequences of dialysis and alternatives, such as renal transplantation and non-dialytic conservative treatments. The discussion should also include potential physical, psychosocial and socioeconomic consequences of each choice. In addition, the patient and family should have time to consider options and clarify doubts, especially before making critical decisions, such as initiating or discontinuing dialysis. At the same time, those involved should be aware that these decisions are open and can be reviewed at any time. Therefore, initiating or discontinuing dialysis should not be considered irrevocable decisions; however, those involved in the initial decision should be advised that this may impose potential limitations on future treatment options.

To establish a minimum threshold of benefits to be achieved by dialysis, below which the sacrifices of initiating or maintaining dialysis are disproportionate or even unacceptable within the sociocultural context, can aid in decision-making. Training in communication and making ethical decisions about offering end-of-life care can help the doctor. It must be borne in mind that futile treatment imposes financial cost and undermines efforts to provide health care to all who need it.

Very young or very old patients, those with multiple comorbidities, patients who reach the medical care already in the terminal stage of the disease, individuals with low educational level and socially and culturally marginalized groups may present barriers to participate in the decision making process. Other limitations include impairment or cognitive immaturity and lack of information on the prognosis of treatment in specific groups of patients.

Therefore, initiating or discontinuing dialysis involves decisions that go beyond the specific action of the medical act. Clinical decision guidelines, especially regarding discontinuation of dialysis, resuscitation orders, and limited time trials of dialysis should be developed to assist the physician and multiprofessional team in facing such situations, without going beyond the limits of responsibility and ethics.

Nephrologists should refer the patient to a supportive service whenever they feel unable to make decisions or to provide for adequate support. For the nephrologist, this implies education and knowledge about shared decisions, advanced care planning, end-of-life counseling, and specific end-of-life medical care. At the same time, other dialysis unit professionals should also be trained to make clinical decisions in a shared manner, including other teams only indirectly involved in dialysis.

## References

[1] Davison SN, Levin A, Moss AH (2015). Executive summary of the KDIGO Controversies Conference on
Supportive Care in Chronic Kidney Disease: developing a roadmap to improving
quality care. Kidney Int.

[2] Moss AH, Ganjoo J, Sharma S (2008). Utility of the "surprise" question to identify dialysis patients
with high mortality. Clin J Am Soc Nephrol.

[3] Cohen LM, Ruthazer R, Moss AH, Germain MJ (2010). Predicting six-month mortality for patients who are on
maintenance hemodialysis. Clin J Am Soc Nephrol.

[4] Oliva JS, Roa LM, Lara A (2013). Survival and factors predicting mortality in hemodialysis
patients over 75 years old. J Nephrol.

[5] Kalantar-Zadeh K, Kilpatrick RD, Kuwae N (2005). Revisiting mortality predictability of serum albumin in the
dialysis population: time dependency, longitudinal changes and
population-attributable fraction. Nephrol Dial Transplant.

[6] Rattanasompattikul M, Feroze U, Molnar MZ (2012). Charlson comorbidity score is a strong predictor of mortality in
hemodialysis patients. Int Urol Nephrol.

[7] Ifudu O, Paul HR, Homel P, Friedman EA (1998). Predictive value of functional status for mortality in patients
on maintenance hemodialysis. Am J Nephrol.

[8] Couchoud C, Labeeuw M, Moranne O, Allot V, Esnault V, Frimat L (2009). A clinical score to predict 6-month prognosis in elderly patients
starting dialysis for end-stage renal disease. Nephrol Dial Transplant.

[9] Approaching death: improving care at the end of life - 1997.

[10] Ellershaw J, Ward C (2003). Care of the dying patient: the last hours or days of
life. BMJ.

[11] Germain M, Cohen LM (2007). Renal supportive care: view from across the pond: the United
States perspective. J Palliat Med.

[12] Brown EA, Chambers EJ, Eggeling C (2008). Palliative care in nephrology. Nephrol Dial Transplant.

[13] Haras M (2008). Planning for a good death: a neglected but essential part of ESRD
care. Nephrol Nurs J.

[14] Moss AH, The Robert Wood Johnson Foundation (2003). Completing the continuum of nephrology care. End Stage Renal disease
Peer Workgroup, recommendations to the field.

[15] Wang D Best practices in palliative care for patients with end stage renal
disease, 2011. Senior Seminar HESA 6380.

[16] Warwick G, Mooney A, Russon L, Hardy R (2013). Planning, initiating and withdrawal of renal replacement
therapy.

[17] WHO (2011). WHO Definition of Palliative Care.

[18] The National Consensus Project for Quality Palliative Care (2013). Clinical Practice Guidelines for Quality Palliative Care.

[19] Sepulveda C, Marlin A, Yoshida T, Ullrich A (2002). Palliative care: the World Health Organization's global
perspective. J Pain Symptom Manage.

[20] Weber A, Jaekel-Reinhard A (2000). Burnout syndrome: a disease of modern societies?. Occup Med.

[21] O'Connor NR, Kumar P (2012). Conservative management of end-stage renal disease without
dialysis: a systematic review. J Palliat Med.

[22] Lee CP, Chertow GM, Zenios SA (2009). An empiric estimate of the value of life: updating the renal
dialysis cost-effectiveness standard. Value Health.

[23] Smith C, Da Silva-Gane M, Chandna S, Warwicker P, Greenwood R, Farrington K (2003). Choosing not to dialyse: evaluation of planned non-dialytic
management in a cohort of patients with end-stage renal
failure. Nephron Clin Pract.

[24] Renal Physicians Association (2010). Shared decision-making in the appropriate initiation of and withdrawal
from dialysis - Clinical Practice Guideline.

[25] Moss AH (2010). Revised dialysis clinical practice guideline promotes more
informed decision-making. Clin J Am Soc Nephrol.

[26] Cohen LM, Germain M, Poppel DM, Woods A, Kjellstrand CM (2000). Dialysis discontinuation and palliative care. Am J Kidney Dis.

[27] Jha V, Martin DE, Bargman JM for the International Society of Nephrology Ethical Dialysis Task Force:
Ethical issues in dialysis therapy.

